# Diagnostic Value of Mean Platelet Volume and Hematological Inflammatory Ratios in Brucellosis: A Case–Control Study

**DOI:** 10.3390/life16020352

**Published:** 2026-02-18

**Authors:** Enes Dalmanoğlu, Yeşim Çağlar, Gülce Eylül Aldemir

**Affiliations:** Department of Infectious Diseases and Clinical Microbiology, Faculty of Medicine, Balıkesir University, Balıkesir 10145, Türkiye; yesim.alpay@balikesir.edu.tr (Y.Ç.); gulce.aldemir@balikesir.edu.tr (G.E.A.)

**Keywords:** brucellosis, mean platelet volume, neutrophil-to-lymphocyte ratio, lymphocyte-to-monocyte ratio, inflammatory biomarkers

## Abstract

Brucellosis diagnosis remains challenging in resource-limited endemic settings. This retrospective case–control study evaluated the diagnostic utility of mean platelet volume (MPV) and hematological inflammatory ratios in brucellosis. Fifty patients with confirmed brucellosis and 50 age-matched healthy controls were included at a university hospital in Turkey (2015–2018). Complete blood count parameters, hematological ratios (neutrophil-to-lymphocyte ratio [NLR], platelet-to-lymphocyte ratio [PLR], lymphocyte-to-monocyte ratio [LMR]), erythrocyte sedimentation rate (ESR), and C-reactive protein (CRP) were measured at diagnosis. Receiver operating characteristic (ROC) curve analysis evaluated diagnostic performance; multivariate logistic regression developed a combined model. Brucellosis patients showed significantly lower MPV (8.04 ± 0.95 vs. 8.56 ± 0.69 fL, *p* = 0.002), higher platelet counts (305.0 ± 116.0 vs. 246.0 ± 55.2 × 10^3^/μL, *p* = 0.002), lower NLR (median: 1.69 vs. 2.07, *p* = 0.013), and higher LMR (median: 5.28 vs. 4.12, *p* = 0.008). ESR demonstrated the best individual diagnostic performance (area under the curve [AUC] = 0.842). The combined model (MPV + ESR + CRP) achieved superior performance (AUC = 0.891, sensitivity 84%, specificity 86%). Limitations include the single-center retrospective design, lack of internal validation, and comparison with healthy controls only. Notably, healthy controls were deliberately selected to establish baseline hematological profiles associated with brucellosis rather than to differentiate it from other infections. Brucellosis presents a unique hematological profile with decreased MPV and altered inflammatory ratios. The combined model offers a potentially cost-effective screening approach for endemic settings, pending external validation.

## 1. Introduction

Brucellosis, caused by intracellular bacteria of the genus *Brucella*, represents one of the most prevalent zoonotic infections worldwide, with over 500,000 new cases reported annually [1,2]. The disease burden is particularly significant in the Mediterranean basin, Middle East, Latin America, and parts of Asia, where it poses substantial challenges to both public health systems and agricultural economies [3,4,5]. In Mediterranean countries such as Lebanon, brucellosis remains a significant One Health concern. Recent studies have reported seroprevalence rates of up to 18.7% in cattle herds in Southern Lebanon, with a documented positive correlation between bovine and human disease burden [6]. Furthermore, regional data indicate that despite control measures, the prevalence in cattle varies significantly by region (up to 13.9% in the Bekaa valley), necessitating comprehensive prevention strategies to mitigate public health risks [7]. Despite advances in diagnostic techniques and therapeutic interventions, brucellosis continues to cause considerable morbidity due to its protean clinical manifestations and tendency for chronicity [8,9].

The pathophysiology of brucellosis involves complex host–pathogen interactions characterized by *Brucella’s* unique ability to survive and replicate within macrophages [10]. This intracellular lifestyle triggers a cascade of inflammatory responses involving both innate and adaptive immunity. The resulting inflammatory milieu is characterized by the production of various cytokines, including interleukin-6 (IL-6), tumor necrosis factor-alpha (TNF-α), and interferon-gamma (IFN-γ), which orchestrate the host’s attempt to control the infection while potentially contributing to tissue damage and clinical manifestations [11,12].

In recent years, there has been growing interest in identifying simple, cost-effective biomarkers that can aid in the diagnosis and monitoring of infectious diseases. Complete blood count (CBC) parameters and their derived ratios have emerged as promising candidates due to their widespread availability, rapid turnaround time, and minimal additional cost [13]. These parameters reflect dynamic changes in circulating blood cells during inflammatory states and may provide valuable insights into disease activity and prognosis [14].

Mean platelet volume (MPV) represents the average size of platelets in circulation and serves as an indicator of platelet activation and function [15]. The seminal work by Robbins and Barnard (1983) first demonstrated that infection induces at least two distinct patterns in platelet parameters: increased platelet count with decreased MPV in non-septic infections, and increased MPV with thrombocytopenia in acute sepsis [16]. Since this foundational observation, MPV has been extensively investigated across a broad spectrum of infectious diseases. In community-acquired pneumonia, Karadag-Oncel et al. (2013) reported that MPV was significantly lower in children with pneumonia compared to healthy controls and could serve as a useful predictor for disease severity [17]. In hepatitis B virus infection, Hu et al. (2014) demonstrated that MPV was significantly increased in chronic and chronic severe hepatitis B patients compared to healthy controls and acute hepatitis B patients, suggesting an association between MPV elevation and disease chronicity [18]. Lee et al. (2015) showed that MPV was significantly elevated in children with acute pyelonephritis compared to lower urinary tract infections, establishing MPV as an independent predictive factor in urinary tract infections [19]. More recently, MPV has been evaluated in orthopedic infections; Paziuk et al. (2020) demonstrated that the platelet count-to-MPV ratio served as a novel adjunct indicator of periprosthetic joint infection, while Muñoz-Mahamud et al. (2022) further validated the role of platelet-related markers including MPV in the diagnosis of chronic periprosthetic joint infection [20,21]. Afyon and Artuk (2016) provided a comprehensive review of MPV across various infectious entities, consolidating evidence that MPV changes are infection-type-dependent and can reflect disease activity and severity [22]. Beyond infections, MPV has demonstrated diagnostic utility in autoimmune diseases such as rheumatoid arthritis, systemic lupus erythematosus, and ankylosing spondylitis [23,24]. During inflammatory states, alterations in MPV can result from various mechanisms, including increased platelet consumption at sites of inflammation, bone marrow suppression by inflammatory cytokines, or compensatory thrombopoiesis [25]. Notably, brucellosis is characterized by a substantial inflammatory burden with elevated levels of interleukin-6 (IL-6), tumor necrosis factor-alpha (TNF-α), and interferon-gamma (IFN-γ), which may directly influence platelet production and morphology [26]. Given these established associations between MPV and inflammatory conditions, investigating MPV in brucellosis—a disease with unique immunopathological features—is scientifically justified. The neutrophil-to-lymphocyte ratio (NLR) has gained recognition as a simple yet informative marker of systemic inflammation, reflecting the balance between innate and adaptive immune responses [27]. Similarly, the platelet-to-lymphocyte ratio (PLR) integrates information about both thrombotic and inflammatory pathways [28]. More recently, the lymphocyte-to-monocyte ratio (LMR) has emerged as a potential biomarker, with studies suggesting its utility in various inflammatory and infectious conditions [29].

Despite the growing body of literature on these hematological indices, their specific roles in brucellosis remain incompletely understood. Limited studies have examined MPV, NLR, PLR, and LMR in brucellosis patients, often with conflicting results and methodological limitations [30,31]. Most existing studies have focused on pediatric populations or specific clinical forms of brucellosis, leaving significant gaps in our understanding of these markers in adult patients with various disease manifestations [32].

The rationale for investigating these markers in brucellosis is multifaceted. First, the often subtle and non-specific presentation of brucellosis can lead to diagnostic delays, particularly in non-endemic areas where clinical suspicion may be low [33]. Simple biomarkers that could aid in early recognition or risk stratification would be valuable additions to the diagnostic armamentarium. Second, in resource-limited settings where specialized serological tests may not be readily available, these routine hematological parameters could provide supportive evidence for clinical decision-making [34,35]. Third, understanding the behavior of these markers in brucellosis could provide insights into the pathophysiological mechanisms underlying the disease and potentially identify targets for therapeutic intervention [36,37].

This study was designed to comprehensively evaluate the diagnostic utility of MPV, NLR, PLR, and LMR in adult patients with brucellosis compared to age-matched healthy controls. The primary objective was to characterize the baseline hematological alterations associated with brucellosis rather than to differentiate it from other infectious diseases. This approach was chosen because, in clinical practice, the initial diagnostic challenge often involves recognizing brucellosis in patients whose routine laboratory workup—including a simple complete blood count—may provide early clues prompting further investigation, particularly in resource-limited endemic settings where specialized serological and microbiological tests may not be readily available. We hypothesized that these readily available hematological indices would demonstrate significant alterations in brucellosis patients, reflecting the underlying inflammatory state, and could potentially serve as adjunctive diagnostic markers. Additionally, we aimed to develop a combined diagnostic model integrating these parameters with traditional inflammatory markers (ESR and CRP), assess their individual and collective diagnostic performance through receiver operating characteristic (ROC) curve analysis, and evaluate their potential for risk stratification and prognosis assessment.

## 2. Materials and Methods

### 2.1. Ethics Approval

This retrospective case–control study was conducted at the Department of Infectious Diseases and Clinical Microbiology, Balıkesir University Faculty of Medicine, Turkey, between January 2015 and December 2018. The study protocol was approved by the Balıkesir University Faculty of Medicine Clinical Research Ethics Committee (Decision No: 2018/28, Date: 31 January 2018). Due to the retrospective nature of the study, the requirement for informed consent was waived by the ethics committee. All procedures were performed in accordance with the ethical standards of the institutional research committee and with the 1964 Helsinki Declaration and its later amendments. The study follows the STROBE (Strengthening the Reporting of Observational Studies in Epidemiology) guidelines for reporting observational studies. The study also adheres to the STARD 2015 (Standards for Reporting Diagnostic Accuracy Studies) guidelines for reporting diagnostic accuracy studies.

### 2.2. Study Population

Fifty patients diagnosed with brucellosis were included in the study. Inclusion criteria were: (1) age ≥18 years; (2) confirmed diagnosis of brucellosis based on compatible clinical symptoms (fever, arthralgia, malaise, night sweats) and either positive blood culture for *Brucella* spp. or standard tube agglutination test (SAT) titer ≥1/160; (3) availability of complete blood count data at diagnosis before treatment initiation; and (4) complete medical records including demographic and clinical data. Patients with SAT titers <1/160 were included only if diagnosis was confirmed by positive blood culture.

Exclusion criteria comprised: (1) concurrent infections or inflammatory conditions; (2) hematological disorders or malignancies; (3) chronic liver disease (Child–Pugh class B or C) or kidney disease (eGFR < 60 mL/min/1.73 m^2^); (4) use of medications affecting blood cell counts (corticosteroids, immunosuppressants, antiplatelet agents) within 30 days prior to diagnosis; (5) pregnancy or lactation; (6) autoimmune diseases; (7) recent surgery or trauma within 3 months; and (8) incomplete laboratory data.

Fifty healthy controls were recruited from individuals attending the hospital for routine health check-ups during the same study period. Controls were age-matched (±5 years) to the patient group. Inclusion criteria for controls were: (1) no acute illness or infectious symptoms in the preceding 3 months; (2) no chronic diseases; (3) no regular medication use; (4) normal complete blood count parameters; (5) normal inflammatory markers (ESR < 20 mm/h for males, <30 mm/h for females; CRP < 5 mg/L); (6) normal liver and kidney function tests; (7) no history of brucellosis or other zoonotic diseases; (8) no occupational livestock exposure in the past year; (9) body mass index between 18.5 and 30 kg/m^2^; and (10) no history of smoking or alcohol abuse.

### 2.3. Data Collection

Data were retrospectively collected from electronic medical records and laboratory information systems using a standardized data collection form. Two independent researchers extracted the data, with discrepancies resolved by consensus with a third researcher. The following information was recorded: demographic data (age, sex, occupation, residence, exposure history); clinical manifestations; physical examination findings; and laboratory parameters.

### 2.4. Laboratory Methods

Complete blood count analysis was performed using Sysmex XN-3000 automated hematology analyzers (Sysmex Corporation, Kobe, Japan) within 2 h of blood collection in K2-EDTA anticoagulated tubes, adhering to recommended preanalytical conditions for reliable MPV measurement [38]. The 2-h time window was selected based on established guidelines indicating optimal MPV stability in K2-EDTA samples after 120 min post-venipuncture, as earlier measurements may be affected by EDTA-induced platelet swelling [39]. The analyzer underwent daily calibration using manufacturer-provided controls with coefficients of variation <3% for all parameters. All blood samples were collected between 8:00 and 10:00 AM to minimize circadian variation effects. Of note, MPV measurements can vary by 20–25% between different analyzer platforms, which should be considered when comparing results across studies [40].

ESR was measured using the Westergren method in accordance with International Council for Standardization in Haematology recommendations. CRP levels were quantified by an immunoturbidimetric assay on a Cobas c501 analyzer (Roche Diagnostics, Mannheim, Germany). The standard tube agglutination test for brucellosis was performed using commercial antigens (Cromatest, Linear Chemicals, Barcelona, Spain), with titers ≥1/160 considered positive. Blood cultures were performed using the BACTEC 9240 automated blood culture system (Becton Dickinson, Sparks, MD, USA) with extended incubation periods up to 21 days.

### 2.5. Calculation of Hematological Ratios

The following ratios were calculated from baseline CBC results: Neutrophil-to-lymphocyte ratio (NLR) = Absolute neutrophil count/Absolute lymphocyte count; Platelet-to-lymphocyte ratio (PLR) = Platelet count/Absolute lymphocyte count; Lymphocyte-to-monocyte ratio (LMR) = Absolute lymphocyte count/Absolute monocyte count.

### 2.6. Sample Size Calculation

Based on preliminary data showing an MPV difference of 0.5 fL with a standard deviation of 0.8 fL between brucellosis patients and controls, a sample size of 41 subjects per group was calculated to achieve 80% power at α = 0.05 (two-tailed) using G*Power 3.1 software. We included 50 subjects per group to account for potential exclusions and increase statistical power.

### 2.7. Statistical Analysis

Statistical analysis was performed using SPSS version 25.0 (IBM Corporation, Armonk, NY, USA) and MedCalc Statistical Software version 20.0 (MedCalc Software Ltd., Ostend, Belgium). Continuous variables are expressed as mean ± standard deviation (SD) for normally distributed data or median (interquartile range [IQR]) for non-normally distributed data. Categorical variables are presented as frequencies and percentages. The normality of the data distribution was assessed using the Shapiro–Wilk test. Comparisons between groups were performed using independent-samples *t*-tests or Welch’s *t*-tests for normally distributed variables, Mann–Whitney U tests for non-normally distributed variables, and Chi-square tests or Fisher’s exact tests for categorical variables.

Receiver operating characteristic (ROC) curve analysis was performed to evaluate diagnostic performance. The area under the curve (AUC) with 95% confidence intervals was calculated using the DeLong method. Pairwise comparison of AUCs between the combined model and individual parameters (ESR alone) was performed using the DeLong test for correlated ROC curves. Optimal cut-off values were determined using the Youden index. Sensitivity, specificity, positive predictive value (PPV), negative predictive value (NPV), positive likelihood ratio (LR+), and negative likelihood ratio (LR−) were calculated for each parameter at the optimal cut-off. Binary logistic regression was performed to develop a combined diagnostic model. Variables with *p* < 0.10 in univariate analysis were included in the multivariate model. Net reclassification improvement (NRI) and integrated discrimination improvement (IDI) were calculated to assess the added value of the combined model.

Given multiple comparisons, Bonferroni correction was applied for the primary hematological parameters with adjusted significance level of *p* < 0.005 (0.05/10 parameters). For inflammatory markers and liver enzymes, a separate Bonferroni correction was applied with *p* < 0.0125. For exploratory hematological ratios (NLR, PLR, LMR), the conventional *p* < 0.05 threshold was maintained without correction. A sensitivity analysis was performed to assess whether biomarker profiles differed between culture-confirmed and serology-confirmed cases, using independent-samples *t*-tests for normally distributed variables and Mann–Whitney U tests for non-normally distributed variables.

## 3. Results

### 3.1. Demographic and Clinical Characteristics

The study included 50 patients with confirmed brucellosis and 50 healthy controls. The groups were well-matched for age and sex distribution. The mean age was 51.0 ± 16.1 years (range: 22–80) in the brucellosis group and 48.0 ± 9.1 years (range: 25–80) in the control group (*p* = 0.282). The sex distribution showed 27 (54%) males in the brucellosis group versus 23 (46%) males in the control group (*p* = 0.420).

Among brucellosis patients, 32 (64%) resided in rural areas and 18 (36%) in urban areas. Occupational livestock exposure was reported in 14 (28%) patients. The most common source of infection was consumption of unpasteurized dairy products (38 patients, 76%), followed by direct animal contact (12 patients, 24%). Clinical manifestations at presentation included arthralgia (25 patients, 50%), malaise (21 patients, 42%), fever (19 patients, 38%), weight loss (17 patients, 34%), night sweats (15 patients, 30%), anorexia (13 patients, 26%), and myalgia (10 patients, 20%). The median duration of symptoms before diagnosis was 4 weeks (range: 1–12 weeks).

Clinical forms of brucellosis were classified as follows: systemic form without focal involvement (36 patients, 72%), osteoarticular involvement (8 patients, 16%), orchitis (3 patients, 6%), lymphadenopathy (2 patients, 4%), and soft tissue involvement (1 patient, 2%).

### 3.2. Laboratory Findings

Table 1 presents the comprehensive comparison of laboratory parameters between brucellosis patients and healthy controls.

### 3.3. Hematological Parameters and Ratios

Brucellosis patients demonstrated significantly higher platelet counts compared to controls (305.0 ± 116.0 vs. 246.0 ± 55.2 × 10^3^/μL, *p* = 0.002). Conversely, MPV values were significantly lower in the patient group (8.04 ± 0.95 vs. 8.56 ± 0.69 fL, *p* = 0.002). The inverse relationship between platelet count and MPV was confirmed by correlation analysis (*r* = −0.31, *p* = 0.027).

The NLR was lower in brucellosis patients compared to controls, with median values of 1.69 (IQR: 1.46–2.10) versus 2.07 (IQR: 1.65–2.89), *p* = 0.013. LMR was significantly higher in brucellosis patients with median values of 5.28 (IQR: 3.84–6.65) versus 4.12 (IQR: 3.15–5.23) in controls, *p* = 0.008. PLR showed no significant difference between groups (148.0 ± 66.9 vs. 160.0 ± 145.0, *p* = 0.603).

### 3.4. Inflammatory Markers

ESR was markedly elevated in brucellosis patients (35.6 ± 26.0 vs. 12.2 ± 11.9 mm/h, *p* < 0.001). CRP levels were also significantly higher in patients with median values of 14.0 mg/L (IQR: 7.8–31.5) versus 3.0 mg/L (IQR: 3.0–4.2) in controls, *p* < 0.001. Among brucellosis patients, 38 (76%) had ESR > 20 mm/h and 35 (70%) had CRP > 10 mg/L.

### 3.5. Serological Findings

Standard tube agglutination test results in brucellosis patients showed the following distribution: 1/80 (11 patients, 22%; all confirmed by positive blood culture), 1/160 (20 patients, 40%), 1/320 (10 patients, 20%), 1/640 (7 patients, 14%), and 1/1280 (2 patients, 4%). Blood cultures were positive in 18 (36%) patients. Higher SAT titers were associated with more severe clinical manifestations and higher inflammatory markers (*p* = 0.018).

Sensitivity analysis comparing culture-confirmed cases (*n* = 18) with serology-confirmed cases (SAT ≥ 1/160, *n* = 32) revealed no significant differences in MPV (7.89 ± 0.98 vs. 8.12 ± 0.93 fL, *p* = 0.412), ESR (38.2 ± 27.4 vs. 34.1 ± 25.3 mm/h, *p* = 0.587), or CRP (median: 16.5 vs. 12.8 mg/L, *p* = 0.324). Similarly, the 11 patients with low SAT titers (1/80) but positive blood cultures showed comparable biomarker profiles to the remaining cohort, supporting the validity of including these cases in the analysis.

### 3.6. ROC Curve Analysis

**Figure 1:**
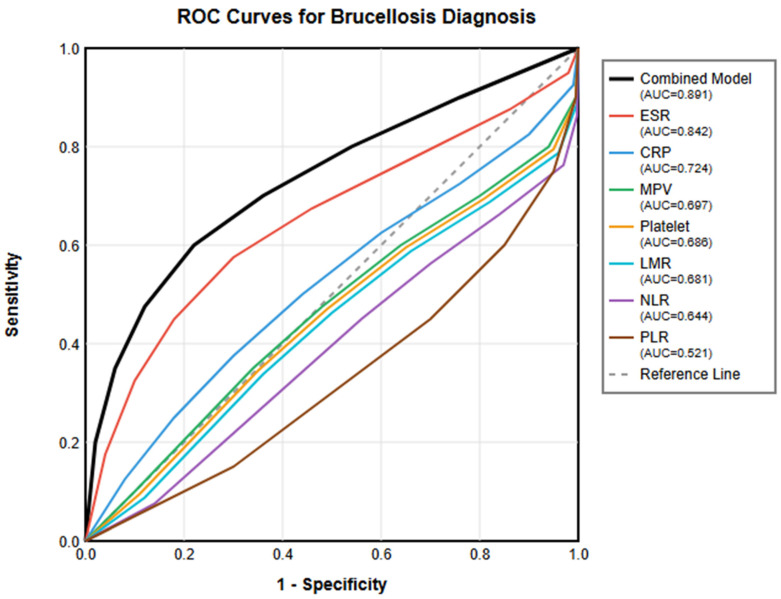
ROC curves showing diagnostic performance of individual parameters (ESR, CRP, MPV, Platelet, LMR, NLR, PLR) and the combined model (MPV + ESR + CRP) for brucellosis diagnosis. The diagonal dashed line represents the reference line (AUC = 0.5).

Comprehensive Receiver operating characteristic (ROC) curve analysis was performed to evaluate the diagnostic performance of all significant parameters (Table 2, Figure 1). ESR demonstrated the best individual diagnostic performance (AUC = 0.842, *p* < 0.001), followed by CRP (AUC = 0.724, *p* < 0.001), MPV (AUC = 0.697, *p* < 0.001), and LMR (AUC = 0.681, *p* = 0.002). PLR showed no significant diagnostic value (AUC = 0.521, *p* = 0.716).

### 3.7. Multivariate Analysis and Combined Diagnostic Model

Binary logistic regression analysis was performed including variables with *p* < 0.10 in univariate analysis. The final model identified ESR (OR = 1.068, 95% CI: 1.035–1.102, *p* < 0.001), CRP (OR = 1.042, 95% CI: 1.015–1.070, *p* = 0.002), and MPV (OR = 0.421, 95% CI: 0.238–0.745, *p* = 0.003) as independent predictors of brucellosis (Table 3). The Hosmer–Lemeshow test indicated good model fit (*p* = 0.652).

The regression equation for the combined model was: Diagnostic Score = −2.147 + (0.065 × ESR) + (0.041 × CRP) − (0.865 × MPV)

The combined model demonstrated superior diagnostic performance (AUC = 0.891, 95% CI: 0.827–0.955, *p* < 0.001) compared to any individual parameter. Pairwise comparison using the DeLong test confirmed that the combined model (AUC = 0.891) significantly outperformed ESR alone (AUC = 0.842; difference = 0.049, z = 2.31, *p* = 0.021). Using a cut-off score of ≥0.5, the model achieved 84% sensitivity and 86% specificity, with PPV of 85.7% and NPV of 84.3%. The net reclassification improvement (NRI) was 0.42 (*p* < 0.001) and the integrated discrimination improvement (IDI) was 0.18 (*p* < 0.001).

### 3.8. Correlation Analysis

Correlation analysis revealed important relationships between parameters (Table 4). ESR showed strong positive correlations with CRP (*r* = 0.56, *p* < 0.001) and moderate correlations with platelet count (*r* = 0.34, *p* = 0.015). MPV demonstrated negative correlations with inflammatory markers: ESR (*r* = −0.30, *p* = 0.035) and CRP (*r* = −0.28, *p* = 0.052). LMR showed negative correlations with both ESR (*r* = −0.32, *p* = 0.024) and CRP (*r* = −0.29, *p* = 0.041).

### 3.9. Subgroup Analysis

When analyzed by clinical form, patients with focal complications (*n* = 14) showed significantly different parameters compared to those with systemic form alone (*n* = 36). Patients with focal complications had higher ESR (48.6 ± 28.2 vs. 30.6 ± 23.4 mm/h, *p* = 0.021), lower MPV (7.62 ± 0.88 vs. 8.21 ± 0.92 fL, *p* = 0.038), and higher diagnostic scores (1.82 ± 0.76 vs. 1.24 ± 0.68, *p* = 0.009).

### 3.10. Treatment Outcomes

Treatment regimens included doxycycline plus rifampin (28 patients, 56%), doxycycline plus streptomycin (12 patients, 24%), doxycycline plus ciprofloxacin (8 patients, 16%), and ciprofloxacin plus rifampin (2 patients, 4%). Complete clinical and laboratory response was achieved in 47 (94%) patients. Three patients (6%) experienced relapse within 6 months of treatment completion. Notably, all relapsed patients had initial MPV values < 7.0 fL and ESR > 50 mm/h. However, given the small number of relapse cases, this observation should be considered preliminary and hypothesis-generating, requiring validation in larger prospective cohorts.

## 4. Discussion

This case–control study provides important insights into the diagnostic utility of hematological inflammatory markers in brucellosis. Our findings reveal a distinct pattern of hematological changes characterized by decreased MPV, increased platelet count, paradoxically lower NLR, and elevated LMR, along with expected elevations in traditional inflammatory markers.

The significantly lower MPV accompanied by reactive thrombocytosis represents a unique hematological signature that distinguishes brucellosis from other bacterial infections. The pathophysiology of reduced MPV in brucellosis involves multiple interconnected mechanisms. The chronic inflammatory environment induced by *Brucella* infection leads to sustained cytokine production, particularly IL-6 and TNF-α, which stimulate thrombopoietin production while simultaneously suppressing megakaryocyte maturation [41]. This results in the production of smaller, less mature platelets despite increased platelet numbers. Additionally, *Brucella* primarily resides within reticuloendothelial system macrophages, creating localized inflammatory foci that continuously consume platelets through formation of platelet–leukocyte aggregates and microthrombi [42].

Our findings align with meta-analysis data by Zhou et al. [43] and Daniels et al. [44], who also reported decreased MPV in brucellosis. However, this contrasts with the elevated MPV typically seen in acute bacterial infections such as sepsis and bacterial meningitis, where MPV values are characteristically increased [45,46]. This discrepancy highlights the unique pathophysiology of brucellosis, where the intracellular lifestyle and ability to evade robust inflammatory responses result in a more chronic, smoldering infection pattern [47]. The decreased MPV pattern observed in brucellosis is more similar to that seen in high-intensity chronic inflammatory conditions such as active rheumatoid arthritis, systemic lupus erythematosus flares, and familial Mediterranean fever relapses, where large activated platelets are preferentially consumed at sites of inflammation, leaving smaller platelets in circulation [48]. This shared pattern suggests that the chronicity and intracellular nature of *Brucella* infection induces a distinct platelet response compared to acute pyogenic infections. Our subgroup analysis revealed that patients with focal complications had even lower MPV values, suggesting that MPV may also reflect disease severity.

When our findings are placed in the broader context of MPV changes across different infectious entities, a nuanced picture emerges. In their landmark study, Robbins and Barnard [16] established that non-septic infections are generally associated with increased platelet count and decreased MPV, whereas acute sepsis triggers the opposite pattern—thrombocytopenia with increased MPV. Our observation of decreased MPV with reactive thrombocytosis in brucellosis is consistent with this foundational paradigm of non-septic infection. A similar pattern of decreased MPV has been reported in community-acquired pneumonia in children [17], supporting the notion that localized or subacute bacterial infections tend to induce smaller platelet volumes through stimulated thrombopoiesis. Conversely, Hu et al. [18] demonstrated that MPV was significantly increased in chronic hepatitis B, particularly in patients with advanced liver disease, reflecting a different pathophysiological mechanism driven by hepatic thrombopoietin dysregulation and portal hypertension rather than a direct infectious inflammatory response. In urinary tract infections, Lee et al. [19] reported elevated MPV in acute pyelonephritis compared to lower urinary tract infections, suggesting that the severity and systemic involvement of infection modulate MPV directionality. Furthermore, in periprosthetic joint infections, Paziuk et al. [20] demonstrated that the platelet count-to-MPV ratio could serve as a diagnostic adjunct, and Muñoz-Mahamud et al. [21] confirmed the potential of platelet-derived markers in chronic orthopedic infections. As comprehensively reviewed by Afyon and Artuk [22], MPV alterations are infection-type-dependent and reflect the interplay between the nature of the pathogen, the chronicity of infection, and the host inflammatory response. In this context, the decreased MPV observed in brucellosis likely reflects the sustained inflammatory stimulus driven by the intracellular persistence of *Brucella* spp., which continuously stimulates megakaryopoiesis and releases a predominance of smaller, newly produced platelets into the circulation.

The paradoxically lower NLR challenges the conventional paradigm of bacterial infection-induced neutrophilia. Unlike pyogenic bacteria, *Brucella* has evolved sophisticated immune evasion strategies. The modified lipopolysaccharide structure contains a diaminoglucose backbone with long-chain fatty acids, reducing TLR4 activation by 100–1000 fold compared to typical Gram-negative bacteria [49]. Additionally, the Type IV secretion system actively suppresses initial neutrophil recruitment [50]. Conversely, the control of brucellosis depends primarily on cell-mediated immunity, with IFN-γ and IL-12 production driving strong Th1 responses [51].

The significantly elevated LMR represents a novel finding not previously reported in the brucellosis literature. This elevation likely reflects the robust lymphocytic response required for controlling intracellular infection, combined with potential monocyte redistribution to infected tissues. The LMR has emerged as a useful biomarker in various conditions, including malignancies and viral infections [52]. The negative correlation between LMR and inflammatory markers suggests that patients with higher LMR may have better immunological control of the infection.

Despite interest in novel hematological ratios, our ROC analysis clearly demonstrated that traditional inflammatory markers remain superior for brucellosis diagnosis. ESR showed the best individual performance, followed by CRP. This superiority likely reflects the direct relationship between these markers and the inflammatory cascade triggered by infection [53]. Interestingly, ESR outperformed CRP, possibly due to its integration of multiple inflammatory factors including fibrinogen, immunoglobulins, and other acute phase proteins [54].

The development of a combined diagnostic model represents a notable finding of our study, though its clinical utility requires validation. By integrating MPV with ESR and CRP, we achieved superior diagnostic accuracy compared to any individual parameter. Although LMR showed significant diagnostic value, it was not included in the final combined model as it did not provide additional discriminatory power beyond that offered by the three selected parameters. The model’s promising performance in this study population suggests it may have potential as an adjunctive screening tool in endemic areas, pending validation against disease controls. The clinical applicability of this model is enhanced by its reliance on routine, inexpensive tests available in most healthcare settings [54].

Our findings have several important clinical implications. First, the recognition of decreased MPV as a characteristic feature of brucellosis adds to the diagnostic clues available to clinicians. In patients presenting with compatible symptoms and decreased MPV in endemic areas, brucellosis should be considered in the differential diagnosis [55]. Second, the combined diagnostic model provides a practical tool for screening and risk stratification. Patients with diagnostic scores ≥0.5 warrant immediate serological testing and consideration for empirical treatment [56]. Third, the association between lower MPV and complicated disease suggests potential for risk stratification, although this requires prospective validation [57]. The economic implications of our diagnostic model are substantial, as in endemic regions, the cost of CBC plus ESR and CRP is significantly lower than that of specific serological tests or blood cultures, enabling risk stratification while preserving expensive confirmatory tests for high-risk patients [58]. Importantly, in brucellosis—a disease that can mimic numerous other conditions and follow a subacute course leading to frequent diagnostic delays—the simultaneous presence of multiple hematological changes (decreased MPV, reactive thrombocytosis, lower NLR, and elevated LMR) in the context of compatible clinical and epidemiological findings may serve as a practical alert for clinicians to pursue confirmatory testing, particularly in settings where advanced diagnostic facilities are unavailable.

Our study has several important limitations that warrant consideration. First, the retrospective design limits causal inference and may introduce selection bias. Second, the single-center nature may affect generalizability to other geographic regions with different epidemiological characteristics. Third, and perhaps most importantly, the diagnostic model was developed and validated on the same dataset without internal validation techniques such as bootstrap resampling or k-fold cross-validation. This approach may result in overfitting and overestimation of diagnostic performance; therefore, the reported AUC of 0.891 should be interpreted as an optimistic estimate. External validation in independent cohorts is essential before clinical implementation.

Fourth, and perhaps most critically, the comparison with healthy controls rather than patients with clinically similar conditions represents a significant methodological limitation. In real clinical practice, the diagnostic challenge lies not in distinguishing brucellosis from healthy individuals but in distinguishing it from other febrile illnesses with overlapping presentations, including typhoid fever, tuberculosis, infective endocarditis, viral infections, and autoimmune diseases such as systemic lupus erythematosus or adult-onset Still’s disease. The observed differences in MPV, NLR, and LMR may reflect nonspecific inflammatory patterns rather than brucellosis-specific signatures. Indeed, decreased MPV has been reported in various inflammatory conditions including active rheumatoid arthritis, lupus flares, and inflammatory bowel disease, limiting the specificity of this finding for brucellosis [48]. The reported sensitivity and specificity values likely represent the upper bounds of what would be achieved in clinical practice, and future studies must include disease controls (febrile patients without brucellosis) to better assess the model’s discriminatory capacity in realistic diagnostic scenarios. However, the deliberate selection of healthy controls in this study served to establish the fundamental hematological profile of brucellosis as a necessary first step before proceeding to comparative studies with disease controls. Furthermore, selecting an appropriate disease control group presents inherent challenges: any single infectious disease comparator would raise questions about why that particular condition was chosen, while including multiple diagnoses would inevitably introduce substantial heterogeneity, potentially obscuring the very patterns we aimed to identify. The present study was not designed to claim that these parameters are entirely specific to brucellosis; rather, we aimed to demonstrate that the constellation of multiple hematological changes, when evaluated alongside the patient’s clinical presentation and epidemiological history, may raise clinical suspicion for brucellosis and prompt further confirmatory testing—even in underdeveloped healthcare settings where only basic laboratory tests such as complete blood count, ESR, and CRP are available.

Fifth, the lack of *Brucella* species identification prevents assessment of species-specific inflammatory responses [59]. In Turkey, *B. melitensis* is the predominant species responsible for human brucellosis, and our results should be interpreted within this epidemiological context. Different *Brucella* species may induce varying degrees of inflammation and distinct hematological profiles, which warrants investigation in future studies. Sixth, the cross-sectional analysis at diagnosis does not capture dynamic changes in markers during treatment, limiting our ability to assess their utility for monitoring therapeutic response. Finally, while we performed sensitivity analysis comparing culture-confirmed and serology-confirmed cases, the inclusion of 11 patients with SAT titers of 1/80 (confirmed by positive blood culture) may have introduced heterogeneity, although our analysis showed comparable biomarker profiles across these subgroups.

Several research directions merit pursuit. Prospective validation of the combined diagnostic model in different populations, including external validation cohorts, would establish broader applicability and provide more reliable estimates of diagnostic performance. Longitudinal studies tracking marker changes during treatment could establish utility for monitoring therapeutic response. Investigation of these markers in different *Brucella* species and comparison with other intracellular pathogens such as *Salmonella typhi*, *Coxiella burnetii*, and *Mycobacterium tuberculosis* would enhance understanding of specificity. Development of point-of-care tests incorporating these parameters could improve diagnosis in resource-limited settings [60,61].

## 5. Conclusions

This study demonstrates that brucellosis induces a distinct hematological profile characterized by decreased MPV, reactive thrombocytosis, paradoxically lower NLR, and elevated LMR, reflecting its unique immunopathology as an intracellular pathogen. The combined diagnostic model incorporating MPV, ESR, and CRP showed promising discriminatory performance, offering a potentially practical and cost-effective adjunctive tool for brucellosis screening in endemic areas. The diagnostic score can be easily calculated using routine laboratory tests. By comparing brucellosis patients with healthy controls, this study establishes the fundamental hematological alterations associated with brucellosis as a necessary foundation for future comparative studies involving disease controls. However, given the limitations of the single-center design, lack of internal validation, and comparison with healthy controls only, these findings should be interpreted as hypothesis-generating. External validation in independent cohorts with disease controls is essential before any clinical implementation can be considered.

## Figures and Tables

**Table 1:** Comparison of Demographic and Laboratory Parameters Between Brucellosis Patients and Controls.

Parameter	Brucellosis (n = 50)	Control (n = 50)	*p*-Value
**Demographics**			
Age (years)	51.0 ± 16.1	48.0 ± 9.1	0.282
Male sex, n (%)	27 (54)	23 (46)	0.420
**Hematological Parameters**			
WBCs (×10^3^/μL)	6.64 ± 1.82	7.03 ± 1.89	0.291
Neutrophils (×10^3^/μL)	3.72 ± 1.49	4.33 ± 1.68	0.054
Lymphocytes (×10^3^/μL)	2.22 ± 0.70	1.95 ± 0.77	0.063
Monocytes (×10^3^/μL)	0.42 ± 0.18	0.48 ± 0.21	0.041
Platelets (×10^3^/μL)	305.0 ± 116.0	246.0 ± 55.2	0.002 *
MPV (fL)	8.04 ± 0.95	8.56 ± 0.69	0.002 *
PDW (%)	16.7 ± 0.59	16.9 ± 0.48	0.143
RDW (%)	14.8 ± 1.81	14.0 ± 2.35	0.070
Hemoglobin (g/dL)	13.0 ± 1.61	13.8 ± 1.64	0.017
Hematocrit (%)	39.3 ± 4.55	40.5 ± 4.46	0.166
**Hematological Ratios**			
NLR †	1.69 (1.46–2.10)	2.07 (1.65–2.89)	0.013
PLR	148.0 ± 66.9	160.0 ± 145.0	0.603
LMR †	5.28 (3.84–6.65)	4.12 (3.15–5.23)	0.008
**Inflammatory Markers**			
ESR (mm/h)	35.6 ± 26.0	12.2 ± 11.9	<0.001 *
CRP (mg/L) †	14.0 (7.8–31.5)	3.0 (3.0–4.2)	<0.001 *
**Liver Enzymes**			
AST (U/L) †	23.0 (18.0–35.0)	19.0 (16.0–24.0)	<0.001 *
ALT (U/L) †	23.0 (17.0–34.0)	18.0 (13.0–26.0)	0.005

Data presented as mean ± SD or † median (IQR) for non-normally distributed variables. * Significant after Bonferroni correction (*p* < 0.005 for hematological parameters, *p* < 0.0125 for inflammatory markers and liver enzymes). Note: Hematological ratios (NLR, PLR, LMR) were analyzed as exploratory outcomes without Bonferroni correction.

**Table 2:** Diagnostic Performance of Laboratory Parameters for Brucellosis.

Parameter	AUC (95% CI)	* p * -Value	Cut-Off	Sensitivity (%)	Specificity (%)	PPV (%)	NPV (%)	LR+	LR−
ESR	0.842 (0.763–0.921)	<0.001	≥20.0 mm/h	76.0	82.0	80.9	77.4	4.22	0.29
CRP	0.724 (0.627–0.821)	<0.001	≥10.0 mg/L	70.0	68.0	68.6	69.4	2.19	0.44
MPV	0.697 (0.596–0.798)	<0.001	≤8.15 fL	64.0	72.0	69.6	66.7	2.29	0.50
LMR	0.681 (0.578–0.784)	0.002	≥4.85	66.0	68.0	67.3	66.7	2.06	0.50
Platelet	0.686 (0.584–0.788)	<0.001	≥265.0 × 10^3^/μL	68.0	66.0	66.7	67.3	2.00	0.48
NLR	0.644 (0.538–0.750)	0.013	≤1.89	60.0	64.0	62.5	61.5	1.67	0.63
PLR	0.521 (0.408–0.634)	0.716	≥148.5	52.0	54.0	52.0	54.0	1.13	0.89

Abbreviations: AUC, area under the curve; CI, confidence interval; PPV, positive predictive value; NPV, negative predictive value; LR+, positive likelihood ratio; LR−, negative likelihood ratio.

**Table 3:** Multivariate Logistic Regression Analysis for Predicting Brucellosis.

Variable	β Coefficient	SE	OR (95% CI)	*p*-Value
ESR	0.065	0.016	1.068 (1.035–1.102)	<0.001
CRP	0.041	0.013	1.042 (1.015–1.070)	0.002
MPV	−0.865	0.290	0.421 (0.238–0.745)	0.003
Constant	−2.147	1.892	-	0.257

Abbreviations: SE, standard error; OR, odds ratio; CI, confidence interval.

**Table 4:** Correlation Matrix of Key Parameters in Brucellosis Patients.

	MPV	NLR	PLR	LMR	PLT	ESR	CRP
MPV	1.00						
NLR	−0.16	1.00					
PLR	−0.24 *	0.41 **	1.00				
LMR	0.22	−0.38 **	−0.45 **	1.00			
PLT	−0.31 *	0.09	0.78 **	−0.18	1.00		
ESR	−0.30 *	0.23	0.19	−0.32 *	0.34 *	1.00	
CRP	−0.28	0.27	0.16	−0.29 *	0.29 *	0.56 **	1.00

* *p* < 0.05, ** *p* < 0.01. Abbreviations: MPV, mean platelet volume; NLR, neutrophil-to-lymphocyte ratio; PLR, platelet-to-lymphocyte ratio; LMR, lymphocyte-to-monocyte ratio; PLT, platelet count; ESR, erythrocyte sedimentation rate; CRP, C-reactive protein.

## Data Availability

The data presented in this study are available on request from the corresponding author.

## Abbreviations

The following abbreviations are used in this manuscript:

AUC	Area under the curve
CBC	Complete blood count
CI	Confidence interval
CRP	C-reactive protein
ESR	Erythrocyte sedimentation rate
IDI	Integrated discrimination improvement
IFN-γ	Interferon-gamma
IL-6	Interleukin-6
IQR	Interquartile range
LMR	Lymphocyte-to-monocyte ratio
LR+	Positive likelihood ratio
LR−	Negative likelihood ratio
MPV	Mean platelet volume
NLR	Neutrophil-to-lymphocyte ratio
NPV	Negative predictive value
NRI	Net reclassification improvement
OR	Odds ratio
PLR	Platelet-to-lymphocyte ratio
PPV	Positive predictive value
ROC	Receiver operating characteristic
SAT	Standard agglutination test
SD	Standard deviation
TNF-α	Tumor necrosis factor-alpha
